# Crystal structures of Zn(cyclam)I_2_ (second monoclinic polymorph) and Zn(cyclam)I(I_3_)

**DOI:** 10.1107/S2056989021011166

**Published:** 2021-10-29

**Authors:** Sergey P. Gavrish, Sergiu Shova, Yaroslaw D. Lampeka

**Affiliations:** a L.V. Pisarzhevskii Institute of Physical Chemistry of the National Academy of Sciences of Ukraine, Prospekt Nauki 31, Kyiv 03028, Ukraine; b "Petru Poni" Institute of Macromolecular Chemistry, Department of Inorganic, Polymers, Aleea Grigore Ghica Voda 41A, RO-700487 Iasi, Romania

**Keywords:** crystal structure, polymorph, cyclam, zinc, iodide, triiodide, hydrogen bonds

## Abstract

The asymmetric unit of the first title compound iodido­(1,4,8,11-tetra­aza­cyclo­tetra­decane-κ^4^ *N* ^1^,*N* ^4^,*N* ^8^,*N* ^11^)zinc(II) iodide, [ZnI(C_10_H_24_N_4_)]I, **I**, consists of the zinc–cyclam macrocyclic cation with one iodide anion coordinated to the metal ion [Zn—I = 2.6619 (5) Å] and the second present as a counter-ion. The asymmetric unit of the second title compound iodido­(1,4,8,11-tetra­aza­cyclo­tetra­decane-κ^4^ *N* ^1^,*N* ^4^,*N* ^8^,*N* ^11^)zinc(II) triiodide, [ZnI(C_10_H_24_N_4_)]I_3_, **II**, consists of half of the centrosymmetric macrocyclic cation, in which the Zn^II^ ion coordinated to an iodide anion [Zn—I = 2.766 (2) Å] is disordered over two positions [Zn⋯Zn = 0.810 (3) Å], and of the two halves of the crystallographically non-equivalent, non-coordinated, centrosymmetric triiodide anions. In both compounds, the *N*,*N*,*N*,*N*-tetra­dentate macrocyclic ligand is present in the most energetically favored *trans*-III conformation. In the crystals of **I**, the [Zn(C_10_H_24_N_4_)I]^+^ cations and the non-coordinated iodide anions are linked by N—H⋯I and bifurcated N—H⋯(I,I) hydrogen bonds, resulting in the formation of two-dimensional networks lying parallel to the (001) and (101) planes. In contrast, the crystals of **II** are built up from infinite chains of the five-coordinate macrocyclic units arranged along the *b*-axis direction and perpendicular sheets formed of the triiodide counter-ions without significant hydrogen bonding between them.

## Abstract 2

The crystal of the first title compound contains five-coordinate [Zn(*L*)I]^+^ (*L* = cyclam) cations and non-coordinated iodide anions; the extended structure is consolidated by N—H⋯I and N—H⋯(I,I) hydrogen bonds. The crystals of the second title compound consist of chains of [Zn(*L*)I]^+^ units and triiodide counter-ions but without significant hydrogen-bonding inter­actions.

## Chemical context

The 14-membered tetra­azamacrocycle 1,4,8,11-tetra­aza­cyclo­tetra­decane (C_10_H_24_N_4_, cyclam, *L*) is one of the most useful and widely studied ligands because of a number of unique properties, such as exceptionally high thermodynamic stability, kinetic inertness and unusual redox properties inherent to its complexes with transition-metal ions (Melson, 1979 ▸; Yatsimirskii & Lampeka, 1985 ▸). Typically, cyclam coordinates to the metal ion by its four N atoms in a planar manner, leaving two vacant *trans* binding sites in the coordination sphere for additional ligands, including halide anions as an important class. To date, a number of complexes of [*M*(*L*)]^2+^ cations (*M* = Cu^II^, Ni^II^, Zn^II^) with halides *X* ^−^ (*X* = Cl, Br, I) have been reported (Ito *et al.*, 1984 ▸; Adam *et al.*, 1991 ▸; Porai-Koshits *et al.*, 1994 ▸; Chen *et al.*, 1996 ▸; Makhaev *et al.*, 1996 ▸; Ha, 2017 ▸; Horii *et al.*, 2020 ▸).

Typically, the compounds under consideration are prepared by the direct reaction of *MX*_2_ salts with *L*. We were inter­ested in the development of alternative methods of synthesizing zinc(II) iodide compounds by anion exchange, starting from the initially formed acetate or nitrate species. It was found in the course of this investigation that precipitation of Zn(*L*)I_2_ from the *in situ* formed acetate complex by potassium iodide in methanol solution occurs slowly (over several days) and results in the formation of the colorless compound **I**, the structure of which is different from that described previously (Porai-Koshits *et al.*, 1994 ▸). When the metathesis reaction was carried out in aqueous solution, a small amount of the iodide/triiodide salt (compound **II**) was obtained in the form of intensely colored brown crystals. The lattice parameters for this compound were reported by Heinlein & Tebbe (1985 ▸) in an alternate setting of the unit cell (see *Database Survey*) but no atomic coordinates were established. Here, we report the crystal structures of these two compounds, namely, iodido-(1,4,8,11-tetra­aza­cyclo­tetra­decane-κ^4^ *N* ^1^ *N* ^4^ *N* ^8^ *N* ^11^)zinc(II) iodide, [ZnI(*L*)]I, **I** and iodido-(1,4,8,11-tetra­aza­cyclo­tetra­decane-κ^4^ *N* ^1^ *N* ^4^ *N* ^8^ *N* ^11^)zinc(II) triiodide, [ZnI(*L*)]I_3_, **II**. 
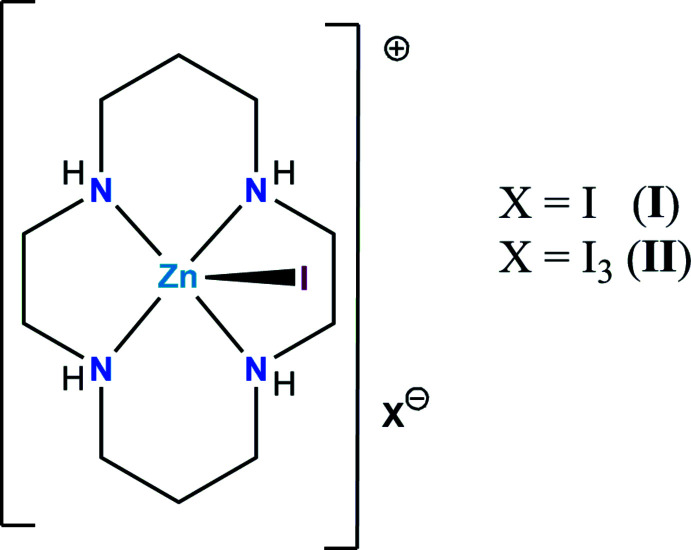


## Structural commentary

**Figure 1:**
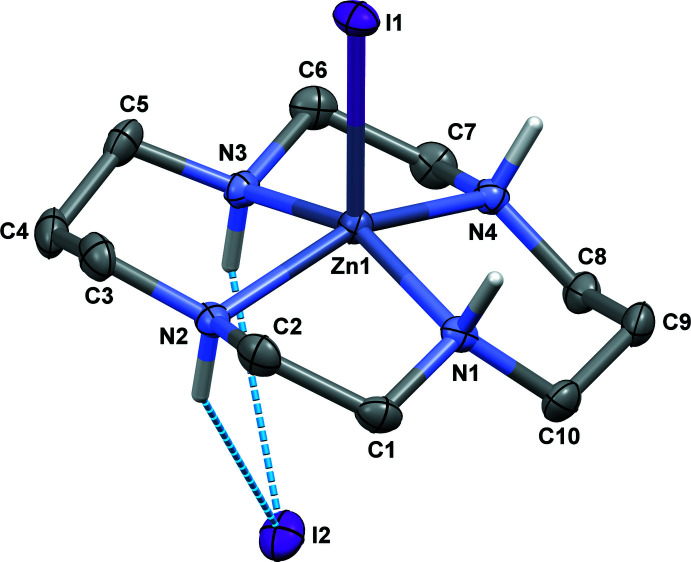
View of the mol­ecular structure of **I** showing the atom-labeling scheme with displacement ellipsoids drawn at the 30% probability level. C-bound H atoms are omitted for clarity. Hydrogen-bonding inter­actions are shown as dashed lines.

The mol­ecular structure of **I** is shown in Fig. 1 ▸. It represents the square-pyramidal macrocyclic [Zn(*L*)I]^+^ cation with one iodide anion coordinated in the axial position of the zinc(II) ion, while the second iodide anion acts as a counter-ion.

Thus, **I** belongs to a rather limited family of [Zn(*L*)] compounds in which the Zn^II^ ion is five-coordinated. Other distinct examples are complexes with thiol­ate (Notni *et al.*, 2006 ▸) and hexa­cyano­ferrate(3–) (Colacio *et al.*, 2001 ▸) axial ligands. In the majority of compounds, the Zn^II^ ion is six-coordinated. Analogously to these complexes, the macrocyclic ligand in **I** adopts the most energetically favorable *trans*-III (*R*,*R*,*S*,*S*) conformation (Bosnich *et al.*, 1965 ▸).

The coordination polyhedron of the [Zn(*L*)I]^+^ cation in **I** is characterized by a large deviation [0.4412 (14) Å] of the metal ion from the mean N_4_ plane of donor atoms toward the coordinated iodide ion and this results in conformational peculiarities, distinguishing it from planar tetra- or hexa-coordinated species. In particular, this deviation results in non-equivalence of the six-membered chelate rings in *chair* conformations with *syn* and *anti* directivity of the NH-hydrogen atoms with respect to the displacement of the metal ion. In the first case, the ring becomes more flattened at the Zn side, and in the second more puckered. Simultaneously, the five-membered rings in **I** adopt *gauche*–*envelope* conformations (one of the carbon atoms lies almost in the N—Zn—N plane) in contrast to the symmetric *gauche* conformations in planar structures.

As expected, the bite angles in the five-membered chelate rings in **I** (*ca* 82.6°, Table 1 ▸) are reduced compared to the typical value of *ca* 85° in planar structures. At the same time, a considerable decrease in the bite angle occurs only in the ‘*anti’* six-membered chelate ring [88.94 (11)° *versus ca* 95° in planar structures].

**Figure 2:**
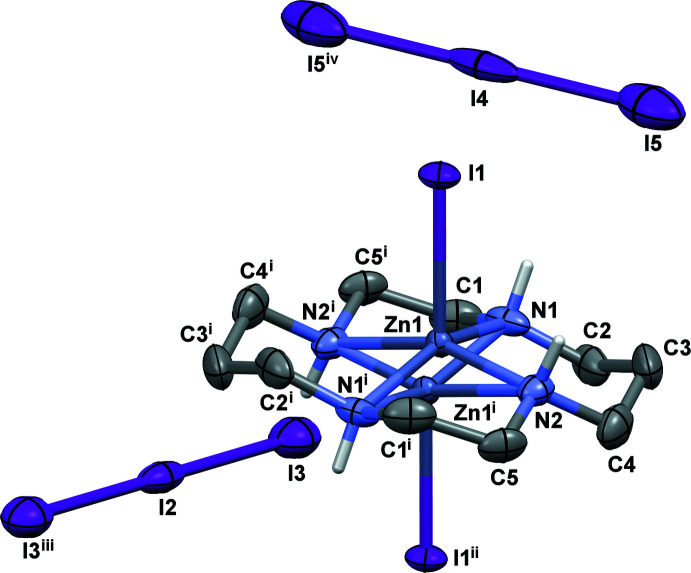
View of the mol­ecular structure of **II** showing the atom-labeling scheme with displacement ellipsoids drawn at the 30% probability level. C-bound H atoms are omitted for clarity. Symmetry codes: (i) −*x* + 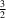 , −*y* + 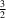 , −*z* + 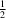 ; (ii) −*x* + 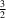 , *y* − 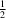 , −*z* + 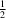 ; (iii) −*x* + 2, *y*, −*z* + 1; (iv) −*x* + 1, *y*, −*z* + 1.]

The mol­ecular structure of compound **II** is shown in Fig. 2 ▸. In this case the [Zn(*L*)] unit is centrosymmetric but the zinc(II) ion is disordered over two positions with site occupancies of 50% constrained by symmetry with a Zn1⋯Zn1^i^ distance of 0.810 (3) Å [symmetry code: (i) −*x* + 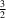 , −*y* + 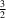 , −*z* + 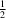 ]. Two crystallographically non-equivalent, non-coord­inated centrosymmetric triiodide anions serve as counter-ions, with I2 and I4 occupying the inversion centers.

The structural characteristics of the [Zn(*L*)I]^+^ unit in **II** are in general agreement with those described above for **I**, with the deviation of the zinc(II) ion from the mean N_4_ plane being 0.381 (2) Å. The ‘*syn’* and ‘*anti’* six-membered chelate rings are characterized by even higher divergences in their bite angles as compared to **I** (10.5° and 6.8°, respectively, Table 1 ▸). The five-membered rings in **II** are also present in *gauche*–*envelope* conformations. A notable distinction in **II** is the considerable difference of the Zn—N bond lengths in the ‘*syn’* and ‘*anti’* six-membered chelate rings [average values = 2.01 (1) and 2.20 (2) Å, respectively], while in **I** this difference is only 0.015 Å.

## Supra­molecular features

**Figure 3:**
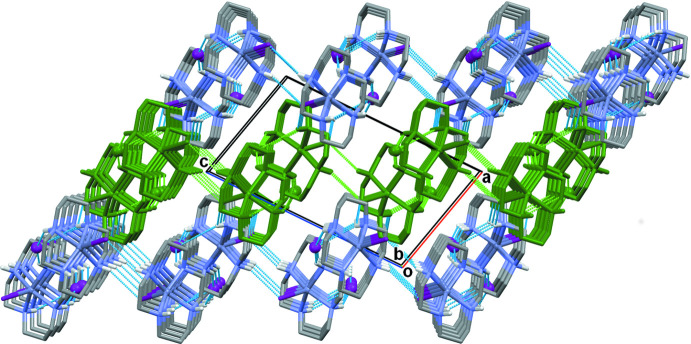
The packing in **I** viewed down the *b*-axis direction. Hydrogen-bonding inter­actions are shown as dashed lines.

**Figure 4:**
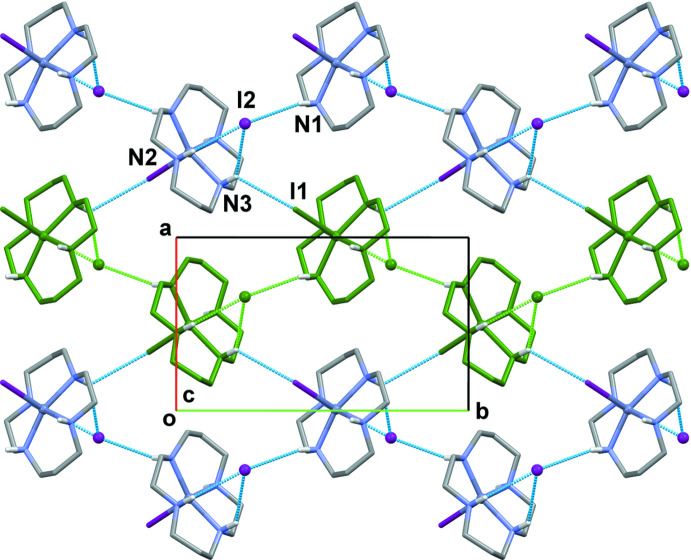
The structure of the hydrogen-bonded layer parallel to the *ab* plane in **I**. Hydrogen-bonding inter­actions are shown as dashed lines.

**Figure 5:**
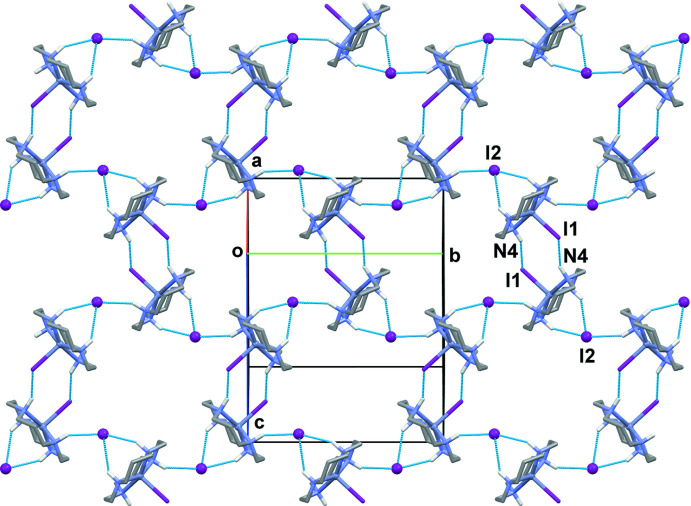
The structure of the hydrogen-bonded layer parallel to the (101) plane in **I**. Hydrogen-bonding inter­actions are shown as dashed lines.

The crystals of **I** have dual lamellar structure. The layers parallel to the *ab* plane are readily discernible (Fig. 3 ▸). They are composed of zigzag chains propagating along the *b*-axis direction, in which the links between the [Zn(*L*)I]^+^ units occur *via* N—H⋯I hydrogen bonds between the secondary amino groups of the macrocyclic ligands (N1—H1, N2—H2 and N3—H3) as the donors and the non-coordinated I2 anions as the acceptors (Table 2 ▸). These chains are linked in the perpendic­ular (*a*-axis) direction through weak N3—H3⋯ I1 bonds (Fig. 4 ▸). At the same time, paired hydrogen-bond contacts involving the coordinated iodide anions I1 and the N4–H4 groups of neighboring macrocycles lead to the formation of another two-dimensional network (Fig. 5 ▸). Since the existence of such hydrogen-bonded layers parallel to the (101) plane is not so evident, one of these sheets in Figs. 3 ▸ and 4 ▸ is highlighted in dark green.

**Figure 6:**
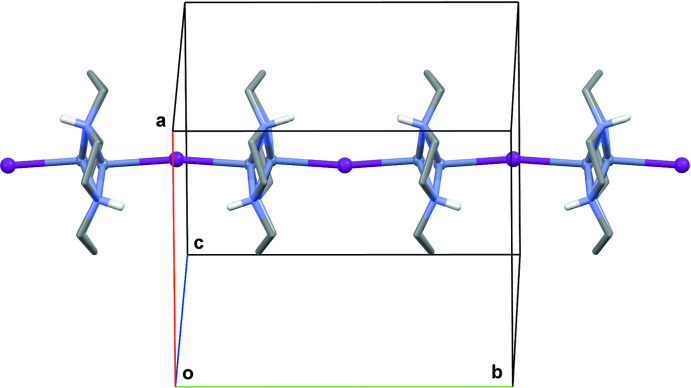
The arrangement of [Zn(*L*)I]^+^ cations along the *b*-axis direction in **II**. C-bound H atoms are omitted for clarity.

**Figure 7:**
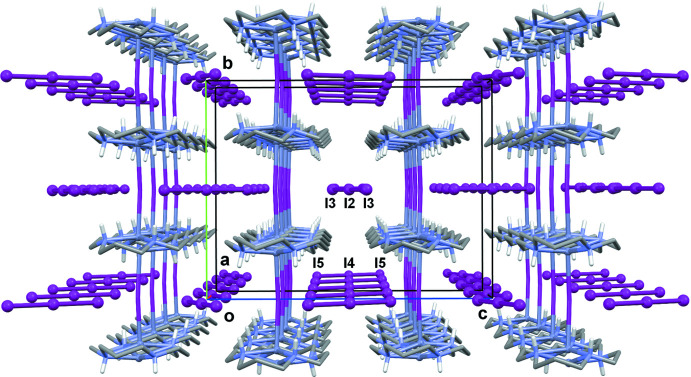
The packing in **II** viewed down the *a*-axis direction. C-bound H atoms are omitted for clarity.

The disordered [Zn(*L*)I]^+^ cations in the crystal of **II** are arranged in parallel chains running along the *b*-axis direction (Fig. 6 ▸). The peculiarity of this structure is that all of the iodine atoms, both coordinated (I1) and those of the triiodide anions [I3/I2/I3^i^ and I5/I4/I5^ii^; symmetry codes (i) −*x* + 2, *y*, −*z* + 1; (ii) −*x* + 1, *y*, −*z* + 1] lie strictly in crystallographic planes parallel to the *ac* plane, thus forming ‘purely iodide’ layers separated by half of the *b* unit-cell length (Fig. 7 ▸). As can be seen, all of the I3—I2—I3 triiodide anions are parallel, as well as the I5—I4—I5 ones, and they form an angle of 71.5 (3)° to each other. The shortest distance between the coordinated iodide and the triiodide anion is 4.803 (3) Å (I1⋯I5), while the shortest distance between triiodide anions is 4.949 (3) Å [I3⋯I3^iii^; symmetry code: (iii) −*x* + 1, *y*, −*z* + 1]. Surprisingly, there are no hydrogen-bonding inter­actions in the crystal of **II** so its three-dimensional structure is based on weak ionic and van der Waals inter­actions.

## Database survey

A search of the Cambridge Structural Database (CSD, version 5.40, last update February 2019; Groom *et al.*, 2016 ▸) indicated that a number of compounds of the composition [*M*(*L*)]*X*_2_ have been characterized structurally. They include complexes of nickel(II) [refcodes TAZDNC01 (Ito *et al.*, 1984 ▸); TAZDNC02–08 (Horii *et al.*, 2020 ▸); RAPKAX (Ha, 2017 ▸); JIZTUH (Adam *et al.*, 1991 ▸); JIZTUH01–04 (Horii *et al.*, 2020 ▸)], copper(II) [TEGPOK (Chen *et al.*, 1996 ▸); TUCQEN (Makhaev *et al.*, 1996 ▸)] and zinc(II) [VUSDUI10, HEGNEM and HEGNOW (Porai-Koshits *et al.*, 1994 ▸)] cyclam cations with the full series (except for Cu*L*Cl_2_) of halide anions (*X* = Cl, Br, I).

In the overwhelming majority of cases, these complexes form monoclinic (space group *P*2_1_/*c* or *P*2_1_/*n*) mol­ecular crystals with the same structural motif: the complex moieties form infinite chains, in which they are joined by the pairs of N—H⋯*X* hydrogen bonds between the NH group of the macrocycle and the coordinated halide ion. On the other hand, in the case of the nickel(II), two other polymorphs of the iodide salt are known. These are also chain structures; however, one of the iodide anions is not coordinated [CAFHUM (Prasad & McAuley, 1983 ▸) and JIZTUH05–08 (Horii *et al.*, 2020 ▸)]. The peculiarity, characteristic only of zinc(II) complexes, is that quite similar to the situation observed in **II**, the metal ion is disordered over two positions. It should also be noted that a degree of pyramidalization of the Zn(N_4_) chromophore progressively increases on going from Cl to I (the deviation of the Zn^II^ ion from the mean N_4_ plane is 0.237, 0.322 and 0.385 Å, respectively) and the conformations of the chelate rings and their bite angles demonstrate systematic trends consistent with this variation. The structure of the complex [Zn(*L*)I]I_3_ is also mentioned (DEHVOB; Heinlein & Tebbe, 1985 ▸), but without atomic coordinates. The reported unit-cell parameters (space group *C*2/*m*; *a* = 19.189, *b* = 12.615, *c* = 10.072 Å; β = 120.65°) represent an alternative setting of the *I*2/*m* unit cell found here for **II**: the matrix 0 0 1 / 0 1 0 / −1 0 1 transforms the DEHVOB cell to that of **II**.

## Synthesis and crystallization

All chemicals and solvents used in this work were purchased from Sigma–Aldrich and were used without further purification.

To prepare **I**, a solution of 48 mg (0.240 mmol) of cyclam in 2 ml of MeOH was added to a solution of 50 mg (0.228 mmol) of Zn(CH_3_CO_2_)_2_·2H_2_O in 2 ml of MeOH and the mixture was heated at *ca* 333 K for 10 h. After cooling, a solution of 0.6 g of KI in 4 ml of MeOH was added and the mixture was left at room temperature. After one week, colorless prismatic crystals formed were filtered off, washed with MeOH and dried in air. Yield: 79 mg (67%). Analysis calculated for C_10_H_24_N_4_Zn_1_I_2_: C 23.12; H 4.66; N 10.78%. Found: C 22.98; H 4.72; N 10.63%. Single crystals of **I** in the form of colorless prisms suitable for X-ray diffraction analysis were picked from the sample resulting from the synthesis.

Crystals of **II** were obtained in an experiment when the precipitation of the product was attempted in aqueous solution. After addition of the solution of 0.5 g of KI in 0.5 ml of H_2_O to the solution of the nitrate salt of the macrocyclic cation [obtained *in situ* from 50 mg (0.25 mmol) of cyclam and 75 mg (0.25 mmol) of Zn(NO_3_)_2_·6H_2_O] in 2 ml of H_2_O, a white precipitate formed (*ca* 92 mg), which was filtered off and the mother liquor was left exposed to the air. After several days, a small qu­antity of brown crystals of **II** had formed, which were picked for crystallographic investigation.

## Refinement

Crystal data, data collection and structure refinement details are summarized in Table 3 ▸. All of the H atoms in **I** and **II** were placed in geometrically idealized positions and constrained to ride on their parent atoms, with C—H = 0.97 Å and N—H = 0.98 Å with U_iso_(H) values of 1.2U_eq_ of the parent atoms.

## Supplementary Material

Crystal structure: contains datablock(s) I, II. DOI: 10.1107/S2056989021011166/hb7994sup1.cif

Structure factors: contains datablock(s) I. DOI: 10.1107/S2056989021011166/hb7994Isup2.hkl

Structure factors: contains datablock(s) II. DOI: 10.1107/S2056989021011166/hb7994IIsup3.hkl

CCDC references: 2115387, 2115388

Additional supporting information:  crystallographic information; 3D view; checkCIF report

## Figures and Tables

**Table 1:** Selected geometrical parameters (Å, °) of the complex cations

**I**		**II**	
Zn1—N1	2.101 (3)	Zn1—N1	2.014 (10)
Zn1—N2	2.121 (3)	Zn1—N2	2.014 (10)
Zn1—N3	2.121 (3)	Zn1—N1i	2.179 (10)
Zn1—N4	2.110 (3)	Zn1—N2i	2.210 (10)
Zn1—I1	2.6619 (5)	Zn1—I1	2.766 (2)
N1—Zn1—N4	95.77 (11)	N1—Zn1—N2	98.9 (5)
N2—Zn1—N3	88.94 (11)	N1^i^—Zn1—N2^i^	88.4 (4)
N1—Zn1—N2	82.64 (11)	N1^i^—Zn1—N2	82.9 (5)
N3—Zn1—N4	82.61 (11)	N1—Zn1—N2^i^	82.2 (5)

Symmetry code: (i) −*x* + {3\over 2}, −*y* + {3\over 2}, −*z* + {1\over 2}.

**Table 2:** Hydrogen-bond geometry (Å, °) for **I** [Chem scheme1]

*D*—H⋯*A*	*D*—H	H⋯*A*	*D*⋯*A*	*D*—H⋯*A*
N1—H1⋯I2^i^	0.98	2.82	3.708 (3)	151
N2—H2⋯I2	0.98	2.78	3.634 (3)	146
N3—H3⋯I1^ii^	0.98	3.20	3.819 (3)	123
N3—H3⋯I2	0.98	3.13	3.897 (3)	137
N4—H4⋯I1^iii^	0.98	2.86	3.680 (3)	142

Symmetry codes: (i) -x-{\script{1\over 2}}, y-{\script{1\over 2}}, -z+{\script{1\over 2}}; (ii) -x+{\script{1\over 2}}, y+{\script{1\over 2}}, -z+{\script{1\over 2}}; (iii) -x, -y+1, -z.

**Table 3:** Experimental details

	**I**	**II**
Crystal data		
Chemical formula	[ZnI(C_10_H_24_N_4_)]I	[ZnI(C_10_H_24_N_4_)]I_3_
*M*_r_	519.50	773.30
Crystal system, space group	Monoclinic, *P*2_1_/*n*	Monoclinic, *I*2/*m*
Temperature (K)	293	293
*a*, *b*, *c* (Å)	8.3837 (3), 13.7570 (4), 14.6478 (5)	10.0629 (12), 12.6263 (12), 16.5133 (16)
β (°)	103.852 (3)	90.921 (10)
*V* (Å^3^)	1640.25 (9)	2097.9 (4)
*Z*	4	4
Radiation type	Mo *K*α	Mo *K*α
μ (mm^−1^)	5.25	7.05
Crystal size (mm)	0.2 × 0.2 × 0.15	0.18 × 0.18 × 0.10

Data collection		
Diffractometer	Xcalibur, Eos	Xcalibur, Eos
Absorption correction	Multi-scan (*CrysAlis PRO*; Rigaku OD, 2019 ▸)	Multi-scan (*CrysAlis PRO*; Rigaku OD, 2019 ▸)
*T*_min_, *T*_max_	0.650, 1.000	0.563, 1.000
No. of measured, independent and observed [*I* > 2σ(*I*)] reflections	10691, 3785, 2982	1931, 1931, 1531
*R*_int_	0.031	0.065
(sin θ/λ)_max_ (Å^−1^)	0.684	0.595

Refinement		
*R*[*F* ^2^ > 2σ(*F* ^2^)], *wR*(*F* ^2^), *S*	0.033, 0.053, 1.02	0.065, 0.209, 1.02
No. of reflections	3785	1931
No. of parameters	155	100
H-atom treatment	H-atom parameters constrained	H-atom parameters constrained
Δρ_max_, Δρ_min_ (e Å^−3^)	0.57, −0.83	1.86, −2.21

Computer programs: *CrysAlis PRO* (Rigaku OD, 2019 ▸), *SHELXT2018/2* (Sheldrick, 2015*a* ▸), *SHELXL2018/3* (Sheldrick, 2015*b* ▸), *Mercury* (Macrae *et al.*, 2020 ▸) and *publCIF* (Westrip, 2010 ▸).

## supplementary crystallographic information

### Iodido(1,4,8,11-tetraazacyclotetradecane-κ4N1,N4,N8,N11)zinc(II) iodide (I) Crystal data

**Table:**

[ZnI(C_10_H_24_N_4_)]I	*F*(000) = 992
*M**_r_* = 519.50	*D*_x_ = 2.104 Mg m^−^^3^
Monoclinic, *P*2_1_/*n*	Mo *K*α radiation, λ = 0.71073 Å
*a* = 8.3837 (3) Å	Cell parameters from 3942 reflections
*b* = 13.7570 (4) Å	θ = 2.1–27.3°
*c* = 14.6478 (5) Å	µ = 5.25 mm^−^^1^
β = 103.852 (3)°	*T* = 293 K
*V* = 1640.25 (9) Å^3^	Prism, clear light colourless
*Z* = 4	0.2 × 0.2 × 0.15 mm

### Iodido(1,4,8,11-tetraazacyclotetradecane-κ4N1,N4,N8,N11)zinc(II) iodide (I) Data collection

**Table:**

Xcalibur, Eos diffractometer	3785 independent reflections
Radiation source: fine-focus sealed X-ray tube, Enhance (Mo) X-ray Source	2982 reflections with *I* > 2σ(*I*)
Graphite monochromator	*R*_int_ = 0.031
Detector resolution: 16.1593 pixels mm^-1^	θ_max_ = 29.1°, θ_min_ = 2.1°
ω scans	*h* = −10→10
Absorption correction: multi-scan (CrysAlisPro; Rigaku OD, 2019)	*k* = −17→11
*T*_min_ = 0.650, *T*_max_ = 1.000	*l* = −18→19
10691 measured reflections	

### Iodido(1,4,8,11-tetraazacyclotetradecane-κ4N1,N4,N8,N11)zinc(II) iodide (I) Refinement

**Table:**

Refinement on *F*^2^	Hydrogen site location: inferred from neighbouring sites
Least-squares matrix: full	H-atom parameters constrained
*R*[*F*^2^ > 2σ(*F*^2^)] = 0.033	*w* = 1/[σ^2^(*F*_o_^2^) + (0.0145*P*)^2^] where *P* = (*F*_o_^2^ + 2*F*_c_^2^)/3
*wR*(*F*^2^) = 0.053	(Δ/σ)_max_ = 0.001
*S* = 1.02	Δρ_max_ = 0.57 e Å^−^^3^
3785 reflections	Δρ_min_ = −0.83 e Å^−^^3^
155 parameters	Extinction correction: *SHELXL2018/3* (Sheldrick 2015b), Fc^*^=kFc[1+0.001xFc^2^λ^3^/sin(2θ)]^-1/4^
0 restraints	Extinction coefficient: 0.00134 (7)
Primary atom site location: structure-invariant direct methods	

### Iodido(1,4,8,11-tetraazacyclotetradecane-κ4N1,N4,N8,N11)zinc(II) iodide (I) Special details

**Table:**

Geometry. All esds (except the esd in the dihedral angle between two l.s. planes) are estimated using the full covariance matrix. The cell esds are taken into account individually in the estimation of esds in distances, angles and torsion angles; correlations between esds in cell parameters are only used when they are defined by crystal symmetry. An approximate (isotropic) treatment of cell esds is used for estimating esds involving l.s. planes.

### Iodido(1,4,8,11-tetraazacyclotetradecane-κ4N1,N4,N8,N11)zinc(II) iodide (I) Fractional atomic coordinates and isotropic or equivalent isotropic displacement parameters (Å^2^)

**Table:**

	*x*	*y*	*z*	*U*_iso_*/*U*_eq_	
I1	0.16199 (3)	0.40650 (2)	0.15018 (2)	0.03711 (9)	
Zn1	0.00746 (5)	0.54540 (3)	0.22192 (3)	0.02665 (12)	
N1	−0.2241 (3)	0.48210 (19)	0.2122 (2)	0.0272 (7)	
H1	−0.226473	0.423178	0.174250	0.033*	
N2	0.0454 (3)	0.5033 (2)	0.36481 (19)	0.0292 (7)	
H2	0.003393	0.556139	0.397386	0.035*	
N3	0.1933 (3)	0.65217 (19)	0.2632 (2)	0.0282 (7)	
H3	0.149659	0.704337	0.295758	0.034*	
N4	−0.0687 (3)	0.63817 (19)	0.1048 (2)	0.0288 (7)	
H4	−0.054780	0.601213	0.050069	0.035*	
C1	−0.2327 (5)	0.4485 (3)	0.3069 (3)	0.0371 (10)	
H1A	−0.306512	0.393370	0.301155	0.044*	
H1B	−0.275717	0.500177	0.339202	0.044*	
C2	−0.0644 (5)	0.4196 (3)	0.3632 (3)	0.0384 (10)	
H2A	−0.068776	0.402191	0.426776	0.046*	
H2B	−0.024607	0.363962	0.334543	0.046*	
C3	0.2174 (5)	0.4875 (3)	0.4148 (3)	0.0410 (10)	
H3A	0.264794	0.437386	0.382861	0.049*	
H3B	0.221722	0.465027	0.478124	0.049*	
C4	0.3177 (5)	0.5806 (3)	0.4193 (3)	0.0463 (11)	
H4A	0.262635	0.631620	0.445670	0.056*	
H4B	0.423918	0.570130	0.462252	0.056*	
C5	0.3457 (4)	0.6162 (3)	0.3260 (3)	0.0415 (10)	
H5A	0.426638	0.667933	0.337714	0.050*	
H5B	0.389197	0.563309	0.295420	0.050*	
C6	0.2140 (5)	0.6907 (3)	0.1731 (3)	0.0389 (10)	
H6A	0.260339	0.641125	0.139970	0.047*	
H6B	0.288015	0.745919	0.183961	0.047*	
C7	0.0475 (5)	0.7213 (3)	0.1152 (3)	0.0389 (10)	
H7A	0.006315	0.775097	0.145756	0.047*	
H7B	0.056870	0.742876	0.053664	0.047*	
C8	−0.2429 (4)	0.6695 (3)	0.0832 (3)	0.0365 (9)	
H8A	−0.267305	0.706063	0.024857	0.044*	
H8B	−0.259879	0.712074	0.132732	0.044*	
C9	−0.3590 (4)	0.5841 (3)	0.0740 (3)	0.0393 (10)	
H9A	−0.323413	0.534611	0.036061	0.047*	
H9B	−0.467479	0.605410	0.040333	0.047*	
C10	−0.3724 (4)	0.5385 (3)	0.1661 (3)	0.0391 (10)	
H10A	−0.389967	0.589381	0.208462	0.047*	
H10B	−0.467193	0.495836	0.154407	0.047*	
I2	−0.15695 (4)	0.73451 (2)	0.37919 (2)	0.04638 (10)	

### Iodido(1,4,8,11-tetraazacyclotetradecane-κ4N1,N4,N8,N11)zinc(II) iodide (I) Atomic displacement parameters (Å^2^)

**Table:**

	*U*^11^	*U*^22^	*U*^33^	*U*^12^	*U*^13^	*U*^23^
I1	0.04629 (18)	0.03129 (16)	0.03725 (17)	0.00582 (11)	0.01687 (13)	−0.00334 (11)
Zn1	0.0257 (2)	0.0263 (2)	0.0277 (3)	−0.00164 (17)	0.00594 (19)	0.00302 (18)
N1	0.0295 (18)	0.0244 (16)	0.0288 (18)	−0.0045 (13)	0.0095 (14)	−0.0032 (13)
N2	0.0333 (18)	0.0282 (17)	0.0252 (18)	0.0054 (13)	0.0054 (14)	−0.0004 (13)
N3	0.0252 (17)	0.0267 (17)	0.0320 (19)	−0.0028 (13)	0.0054 (14)	−0.0032 (13)
N4	0.0294 (17)	0.0297 (17)	0.0276 (18)	−0.0024 (13)	0.0073 (14)	−0.0020 (13)
C1	0.039 (2)	0.037 (2)	0.039 (3)	−0.0065 (18)	0.019 (2)	−0.0012 (19)
C2	0.048 (3)	0.037 (2)	0.032 (2)	−0.0008 (19)	0.014 (2)	0.0108 (18)
C3	0.050 (3)	0.039 (3)	0.030 (2)	0.0088 (19)	0.001 (2)	0.0068 (18)
C4	0.039 (3)	0.051 (3)	0.039 (3)	0.009 (2)	−0.010 (2)	−0.007 (2)
C5	0.025 (2)	0.040 (2)	0.054 (3)	−0.0029 (17)	−0.002 (2)	−0.005 (2)
C6	0.042 (3)	0.029 (2)	0.048 (3)	−0.0098 (18)	0.016 (2)	−0.0040 (19)
C7	0.050 (3)	0.026 (2)	0.042 (3)	−0.0054 (18)	0.014 (2)	0.0059 (18)
C8	0.037 (2)	0.036 (2)	0.034 (2)	0.0092 (17)	0.0049 (18)	0.0057 (18)
C9	0.027 (2)	0.048 (3)	0.037 (3)	0.0015 (18)	−0.0034 (18)	−0.0012 (19)
C10	0.025 (2)	0.047 (3)	0.044 (3)	−0.0047 (18)	0.0070 (19)	−0.003 (2)
I2	0.04667 (19)	0.03273 (17)	0.0554 (2)	0.00644 (12)	0.00368 (14)	0.00011 (13)

### Iodido(1,4,8,11-tetraazacyclotetradecane-κ4N1,N4,N8,N11)zinc(II) iodide (I) Geometric parameters (Å, º)

**Table:**

I1—Zn1	2.6619 (5)	C3—H3A	0.9700
Zn1—N1	2.101 (3)	C3—H3B	0.9700
Zn1—N2	2.121 (3)	C3—C4	1.525 (5)
Zn1—N3	2.121 (3)	C4—H4A	0.9700
Zn1—N4	2.110 (3)	C4—H4B	0.9700
N1—H1	0.9800	C4—C5	1.522 (6)
N1—C1	1.481 (4)	C5—H5A	0.9700
N1—C10	1.483 (4)	C5—H5B	0.9700
N2—H2	0.9800	C6—H6A	0.9700
N2—C2	1.471 (4)	C6—H6B	0.9700
N2—C3	1.468 (4)	C6—C7	1.509 (5)
N3—H3	0.9800	C7—H7A	0.9700
N3—C5	1.471 (4)	C7—H7B	0.9700
N3—C6	1.470 (4)	C8—H8A	0.9700
N4—H4	0.9800	C8—H8B	0.9700
N4—C7	1.486 (4)	C8—C9	1.510 (5)
N4—C8	1.482 (4)	C9—H9A	0.9700
C1—H1A	0.9700	C9—H9B	0.9700
C1—H1B	0.9700	C9—C10	1.516 (5)
C1—C2	1.507 (5)	C10—H10A	0.9700
C2—H2A	0.9700	C10—H10B	0.9700
C2—H2B	0.9700		
			
N1—Zn1—I1	101.81 (8)	N2—C3—H3B	109.4
N1—Zn1—N2	82.64 (11)	N2—C3—C4	111.3 (3)
N1—Zn1—N3	155.51 (11)	H3A—C3—H3B	108.0
N1—Zn1—N4	95.77 (11)	C4—C3—H3A	109.4
N2—Zn1—I1	102.79 (8)	C4—C3—H3B	109.4
N2—Zn1—N3	88.94 (11)	C3—C4—H4A	108.4
N3—Zn1—I1	102.47 (8)	C3—C4—H4B	108.4
N4—Zn1—I1	101.21 (8)	H4A—C4—H4B	107.4
N4—Zn1—N2	155.76 (11)	C5—C4—C3	115.7 (3)
N4—Zn1—N3	82.61 (11)	C5—C4—H4A	108.4
Zn1—N1—H1	105.9	C5—C4—H4B	108.4
C1—N1—Zn1	108.5 (2)	N3—C5—C4	111.9 (3)
C1—N1—H1	105.9	N3—C5—H5A	109.2
C1—N1—C10	111.5 (3)	N3—C5—H5B	109.2
C10—N1—Zn1	118.3 (2)	C4—C5—H5A	109.2
C10—N1—H1	105.9	C4—C5—H5B	109.2
Zn1—N2—H2	107.0	H5A—C5—H5B	107.9
C2—N2—Zn1	104.6 (2)	N3—C6—H6A	110.1
C2—N2—H2	107.0	N3—C6—H6B	110.1
C3—N2—Zn1	115.5 (2)	N3—C6—C7	108.2 (3)
C3—N2—H2	107.0	H6A—C6—H6B	108.4
C3—N2—C2	115.3 (3)	C7—C6—H6A	110.1
Zn1—N3—H3	108.1	C7—C6—H6B	110.1
C5—N3—Zn1	114.5 (2)	N4—C7—C6	109.7 (3)
C5—N3—H3	108.1	N4—C7—H7A	109.7
C6—N3—Zn1	103.3 (2)	N4—C7—H7B	109.7
C6—N3—H3	108.1	C6—C7—H7A	109.7
C6—N3—C5	114.3 (3)	C6—C7—H7B	109.7
Zn1—N4—H4	106.2	H7A—C7—H7B	108.2
C7—N4—Zn1	108.6 (2)	N4—C8—H8A	109.2
C7—N4—H4	106.2	N4—C8—H8B	109.2
C8—N4—Zn1	116.1 (2)	N4—C8—C9	112.0 (3)
C8—N4—H4	106.2	H8A—C8—H8B	107.9
C8—N4—C7	112.7 (3)	C9—C8—H8A	109.2
N1—C1—H1A	109.6	C9—C8—H8B	109.2
N1—C1—H1B	109.6	C8—C9—H9A	108.5
N1—C1—C2	110.3 (3)	C8—C9—H9B	108.5
H1A—C1—H1B	108.1	C8—C9—C10	115.2 (3)
C2—C1—H1A	109.6	H9A—C9—H9B	107.5
C2—C1—H1B	109.6	C10—C9—H9A	108.5
N2—C2—C1	107.6 (3)	C10—C9—H9B	108.5
N2—C2—H2A	110.2	N1—C10—C9	112.9 (3)
N2—C2—H2B	110.2	N1—C10—H10A	109.0
C1—C2—H2A	110.2	N1—C10—H10B	109.0
C1—C2—H2B	110.2	C9—C10—H10A	109.0
H2A—C2—H2B	108.5	C9—C10—H10B	109.0
N2—C3—H3A	109.4	H10A—C10—H10B	107.8
			
Zn1—N1—C1—C2	−30.6 (3)	N4—C8—C9—C10	76.8 (4)
Zn1—N1—C10—C9	46.4 (4)	C1—N1—C10—C9	173.2 (3)
Zn1—N2—C2—C1	−50.4 (3)	C2—N2—C3—C4	−175.4 (3)
Zn1—N2—C3—C4	62.3 (4)	C3—N2—C2—C1	−178.3 (3)
Zn1—N3—C5—C4	−63.1 (4)	C3—C4—C5—N3	69.4 (4)
Zn1—N3—C6—C7	53.0 (3)	C5—N3—C6—C7	178.0 (3)
Zn1—N4—C7—C6	27.4 (3)	C6—N3—C5—C4	178.0 (3)
Zn1—N4—C8—C9	−53.1 (4)	C7—N4—C8—C9	−179.3 (3)
N1—C1—C2—N2	55.6 (4)	C8—N4—C7—C6	157.5 (3)
N2—C3—C4—C5	−68.4 (4)	C8—C9—C10—N1	−72.4 (4)
N3—C6—C7—N4	−55.5 (4)	C10—N1—C1—C2	−162.5 (3)

### Iodido(1,4,8,11-tetraazacyclotetradecane-κ4N1,N4,N8,N11)zinc(II) iodide (I) Hydrogen-bond geometry (Å, º)

**Table:**

*D*—H···*A*	*D*—H	H···*A*	*D*···*A*	*D*—H···*A*
N1—H1···I2^i^	0.98	2.82	3.708 (3)	151
N2—H2···I2	0.98	2.78	3.634 (3)	146
N3—H3···I1^ii^	0.98	3.20	3.819 (3)	123
N3—H3···I2	0.98	3.13	3.897 (3)	137
N4—H4···I1^iii^	0.98	2.86	3.680 (3)	142

Symmetry codes: (i) −*x*−1/2, *y*−1/2, −*z*+1/2; (ii) −*x*+1/2, *y*+1/2, −*z*+1/2; (iii) −*x*, −*y*+1, −*z*.

### Iodido(1,4,8,11-tetraazacyclotetradecane-κ4N1,N4,N8,N11)zinc(II) triiodide (II) Crystal data

**Table:**

[ZnI(C_10_H_24_N_4_)]I_3_	*F*(000) = 1416
*M**_r_* = 773.30	*D*_x_ = 2.448 Mg m^−^^3^
Monoclinic, *I*2/*m*	Mo *K*α radiation, λ = 0.71073 Å
*a* = 10.0629 (12) Å	Cell parameters from 2913 reflections
*b* = 12.6263 (12) Å	θ = 2.0–26.7°
*c* = 16.5133 (16) Å	µ = 7.05 mm^−^^1^
β = 90.921 (10)°	*T* = 293 K
*V* = 2097.9 (4) Å^3^	Prism, clear light red
*Z* = 4	0.18 × 0.18 × 0.10 mm

### Iodido(1,4,8,11-tetraazacyclotetradecane-κ4N1,N4,N8,N11)zinc(II) triiodide (II) Data collection

**Table:**

Xcalibur, Eos diffractometer	1931 independent reflections
Radiation source: fine-focus sealed X-ray tube, Enhance (Mo) X-ray Source	1531 reflections with *I* > 2σ(*I*)
Graphite monochromator	*R*_int_ = 0.065
Detector resolution: 16.1593 pixels mm^-1^	θ_max_ = 25.0°, θ_min_ = 2.0°
ω scans	*h* = −11→11
Absorption correction: multi-scan (CrysAlisPro; Rigaku OD, 2019)	*k* = −14→15
*T*_min_ = 0.563, *T*_max_ = 1.000	*l* = −19→19
1931 measured reflections	

### Iodido(1,4,8,11-tetraazacyclotetradecane-κ4N1,N4,N8,N11)zinc(II) triiodide (II) Refinement

**Table:**

Refinement on *F*^2^	Primary atom site location: dual
Least-squares matrix: full	Hydrogen site location: mixed
*R*[*F*^2^ > 2σ(*F*^2^)] = 0.065	H-atom parameters constrained
*wR*(*F*^2^) = 0.209	*w* = 1/[σ^2^(*F*_o_^2^) + (0.1207*P*)^2^ + 24.1639*P*] where *P* = (*F*_o_^2^ + 2*F*_c_^2^)/3
*S* = 1.02	(Δ/σ)_max_ < 0.001
1931 reflections	Δρ_max_ = 1.86 e Å^−^^3^
100 parameters	Δρ_min_ = −2.20 e Å^−^^3^
0 restraints	

### Iodido(1,4,8,11-tetraazacyclotetradecane-κ4N1,N4,N8,N11)zinc(II) triiodide (II) Special details

**Table:**

Geometry. All esds (except the esd in the dihedral angle between two l.s. planes) are estimated using the full covariance matrix. The cell esds are taken into account individually in the estimation of esds in distances, angles and torsion angles; correlations between esds in cell parameters are only used when they are defined by crystal symmetry. An approximate (isotropic) treatment of cell esds is used for estimating esds involving l.s. planes.
Refinement. Refined as a 2-component twin.

### Iodido(1,4,8,11-tetraazacyclotetradecane-κ4N1,N4,N8,N11)zinc(II) triiodide (II) Fractional atomic coordinates and isotropic or equivalent isotropic displacement parameters (Å^2^)

**Table:**

	*x*	*y*	*z*	*U*_iso_*/*U*_eq_	Occ. (<1)
I1	0.75984 (13)	1.000000	0.25422 (8)	0.0546 (4)	
Zn1	0.7426 (3)	0.78147 (17)	0.24844 (16)	0.0313 (6)	0.5
N1	0.5759 (11)	0.7637 (8)	0.3129 (7)	0.054 (3)	
H1	0.547169	0.831842	0.316695	0.065*	
N2	0.6627 (11)	0.7691 (9)	0.1362 (6)	0.052 (2)	
H2	0.638693	0.836861	0.124367	0.062*	
C1	0.5986 (19)	0.7286 (12)	0.3953 (8)	0.071 (4)	
H1A	0.592616	0.652075	0.398272	0.085*	
H1B	0.532274	0.758792	0.430502	0.085*	
C2	0.4593 (13)	0.7157 (13)	0.2742 (11)	0.071 (4)	
H2A	0.382036	0.730667	0.306679	0.085*	
H2B	0.471137	0.639494	0.272985	0.085*	
C3	0.4330 (15)	0.7558 (16)	0.1869 (12)	0.088 (6)	
H3A	0.344086	0.734576	0.170108	0.106*	
H3B	0.435683	0.832586	0.187081	0.106*	
C4	0.5303 (16)	0.7154 (15)	0.1257 (9)	0.076 (5)	
H4A	0.541286	0.639577	0.132177	0.091*	
H4B	0.495758	0.728671	0.071478	0.091*	
C5	0.7596 (16)	0.7338 (12)	0.0779 (7)	0.063 (4)	
H5A	0.737529	0.762622	0.024916	0.076*	
H5B	0.757810	0.657158	0.074099	0.076*	
I2	1.000000	0.500000	0.500000	0.0780 (7)	
I3	0.7276 (2)	0.500000	0.55899 (13)	0.0992 (7)	
I4	0.500000	1.000000	0.500000	0.1262 (15)	
I5	0.3190 (3)	1.000000	0.3615 (2)	0.1519 (13)	

### Iodido(1,4,8,11-tetraazacyclotetradecane-κ4N1,N4,N8,N11)zinc(II) triiodide (II) Atomic displacement parameters (Å^2^)

**Table:**

	*U*^11^	*U*^22^	*U*^33^	*U*^12^	*U*^13^	*U*^23^
I1	0.0706 (8)	0.0283 (6)	0.0650 (8)	0.000	0.0056 (6)	0.000
Zn1	0.0299 (11)	0.0357 (16)	0.0285 (10)	0.0018 (15)	0.0024 (8)	0.0004 (15)
N1	0.061 (6)	0.032 (5)	0.070 (7)	0.005 (5)	0.028 (5)	0.001 (5)
N2	0.067 (6)	0.041 (6)	0.048 (5)	−0.005 (5)	−0.008 (5)	0.002 (4)
C1	0.109 (12)	0.056 (8)	0.049 (7)	−0.003 (9)	0.028 (8)	0.002 (6)
C2	0.036 (6)	0.062 (9)	0.115 (13)	−0.002 (6)	0.017 (7)	0.003 (9)
C3	0.049 (8)	0.084 (13)	0.131 (16)	0.014 (8)	−0.031 (10)	−0.002 (11)
C4	0.084 (10)	0.078 (11)	0.064 (9)	−0.008 (9)	−0.034 (8)	0.009 (8)
C5	0.104 (11)	0.056 (9)	0.030 (6)	−0.004 (8)	0.010 (7)	−0.004 (5)
I2	0.1383 (19)	0.0391 (10)	0.0555 (10)	0.000	−0.0321 (11)	0.000
I3	0.1264 (15)	0.0671 (11)	0.1039 (13)	0.000	−0.0103 (11)	0.000
I4	0.124 (2)	0.0324 (10)	0.227 (4)	0.000	0.123 (2)	0.000
I5	0.1283 (19)	0.0718 (13)	0.258 (4)	0.000	0.062 (2)	0.000

### Iodido(1,4,8,11-tetraazacyclotetradecane-κ4N1,N4,N8,N11)zinc(II) triiodide (II) Geometric parameters (Å, º)

**Table:**

I1—Zn1^i^	2.766 (2)	N2—C4	1.502 (19)
I1—Zn1	2.766 (2)	N2—C5	1.451 (17)
Zn1—Zn1^ii^	0.810 (4)	C1—C5^ii^	1.56 (2)
Zn1—N1^ii^	2.179 (10)	C2—C3	1.55 (2)
Zn1—N1	2.014 (10)	C3—C4	1.51 (3)
Zn1—N2^ii^	2.210 (10)	I2—I3^iii^	2.924 (2)
Zn1—N2	2.014 (10)	I2—I3	2.924 (2)
N1—C1	1.444 (19)	I4—I5^iv^	2.901 (4)
N1—C2	1.458 (19)	I4—I5	2.901 (4)
			
Zn1^i^—I1—Zn1	171.86 (12)	C1—N1—Zn1^ii^	103.7 (9)
Zn1^ii^—Zn1—I1	164.9 (4)	C1—N1—C2	113.5 (12)
Zn1^ii^—Zn1—N1^ii^	67.6 (4)	C2—N1—Zn1^ii^	111.2 (9)
Zn1^ii^—Zn1—N1	90.6 (5)	C2—N1—Zn1	119.1 (9)
Zn1^ii^—Zn1—N2	93.0 (5)	Zn1—N2—Zn1^ii^	21.47 (16)
Zn1^ii^—Zn1—N2^ii^	65.5 (4)	C4—N2—Zn1	118.8 (9)
N1^ii^—Zn1—I1	103.0 (3)	C4—N2—Zn1^ii^	109.8 (8)
N1—Zn1—I1	98.3 (3)	C5—N2—Zn1^ii^	101.5 (8)
N1—Zn1—N1^ii^	158.18 (16)	C5—N2—Zn1	111.8 (8)
N1—Zn1—N2^ii^	82.2 (5)	C5—N2—C4	112.9 (12)
N1^ii^—Zn1—N2^ii^	88.4 (4)	N1—C1—C5^ii^	107.7 (11)
N2^ii^—Zn1—I1	103.4 (3)	N1—C2—C3	113.4 (13)
N2—Zn1—I1	97.6 (3)	C4—C3—C2	114.3 (13)
N2—Zn1—N1	98.9 (5)	N2—C4—C3	110.8 (13)
N2—Zn1—N1^ii^	82.9 (5)	N2—C5—C1^ii^	109.8 (10)
N2—Zn1—N2^ii^	158.53 (16)	I3^iii^—I2—I3	180.0
Zn1—N1—Zn1^ii^	21.82 (16)	I5^iv^—I4—I5	180.0
C1—N1—Zn1	114.3 (10)		

Symmetry codes: (i) *x*, −*y*+2, *z*; (ii) −*x*+3/2, −*y*+3/2, −*z*+1/2; (iii) −*x*+2, −*y*+1, −*z*+1; (iv) −*x*+1, −*y*+2, −*z*+1.
